# Contact mechanics of modular metal-on-polyethylene total hip replacement under adverse edge loading conditions

**DOI:** 10.1016/j.jbiomech.2014.08.015

**Published:** 2014-10-17

**Authors:** Xijin Hua, Junyan Li, Ling Wang, Zhongmin Jin, Ruth Wilcox, John Fisher

**Affiliations:** a Institute of Medical and Biological Engineering, School of Mechanical Engineering, University of Leeds, Leeds, UK; b State Key Laboratory for Manufacturing System Engineering, Xi’an Jiaotong University, Xi’an, Shanxi, China

**Keywords:** Microseparation, Edge loading, Metal-on-polyethylene, Contact mechanics, Total hip replacement

## Abstract

Edge loading can negatively impact the biomechanics and long-term performance of hip replacements. Although edge loading has been widely investigated for hard-on-hard articulations, limited work has been conducted for hard-on-soft combinations. The aim of the present study was to investigate edge loading and its effect on the contact mechanics of a modular metal-on-polyethylene (MoP) total hip replacement (THR). A three-dimensional finite element model was developed based on a modular MoP bearing. Different cup inclination angles and head lateral microseparation were modelled and their effect on the contact mechanics of the modular MoP hip replacement were examined. The results showed that lateral microseparation caused loading of the head on the rim of the cup, which produced substantial increases in the maximum von Mises stress in the polyethylene liner and the maximum contact pressure on both the articulating surface and backside surface of the liner. Plastic deformation of the liner was observed under both standard conditions and microseparation conditions, however, the maximum equivalent plastic strain in the liner under microseparation conditions of 2000 µm was predicted to be approximately six times that under standard conditions. The study has indicated that correct positioning the components to avoid edge loading is likely to be important clinically even for hard-on-soft bearings for THR.

## 1 Introduction

Hip joint replacements have been successfully used in orthopaedics for over fifty years. Whilst clinical studies have shown encouraging long-term clinical performance, failure of these devices can still occur. Specifically, the clinical complications and unexpected failure of hip prostheses linked to edge loading have been reported recently (Langton et al., 2011, Walter et al., 2011, Kwon et al., 2012). This edge loading, defined as the contact of the head on the rim of the liner, has been associated with many factors, including patient activity, prosthesis design, surgical positioning and material combinations (Mellon et al., 2011, Wang et al., 2012, Harris, 2012, Elkins et al., 2012; Harris, 2012; Underwood et al., 2012). In particular, the primary contribution of the rotational and translational mal-positioning of the components to edge loading has been identified and well summarised (Fisher, 2011, Harris, 2012). Rotational mal-positioning is defined clinically as the steep inclination and excessive anteversion of the acetabular component while translational mal-positioning, also termed as microseparation, is described as the misalignment of the centres of the head and the cup (Nevelos et al., 1999, Nevelos et al., 2000).

*In vitro* studies have shown that the introduction of microseparation in a hip joint simulator can successfully reproduce clinically relevant wear rates, wear patterns and wear particle distributions for both metal-on-metal (MoM) and ceramic-on-ceramic (CoC) articulations (Nevelos et al., 2000, Stewart et al., 2001). These outcomes, however, could not be replicated under standard walking conditions with either a normal or a steep cup angle (Williams et al., 2008, Angadji et al., 2009). This indicates that microseparation of the femoral head and the acetabular cup occurs *in vivo* during normal gait, a phenomenon which has also been observed with the aid of fluoroscopy (Dennis et al., 2001, Komistek et al., 2002).

Microseparation usually occurs during the swing phase and is considered to be as a result of muscle weakness, mal-positioning of the acetabular cup or offset deficiency of the femoral head (Ryou et al., 2004). These factors cause the femoral head to be moved laterally relative to the acetabular cup during the swing phase. When a load is applied in the stance phase, the femoral head is moved upward, leading to edge loading, which can have a significant consequence on the wear and biomechanics of the total hip replacements (THRs).

The effect of edge loading induced by microseparation on the biomechanics and performance of hard-on-hard articulations has been documented (Manaka et al., 2004, Williams et al., 2006, Leslie et al., 2009, Al-Hajjar et al., 2010). In MoM articulations, edge loading can produce accelerated wear of whole joints (Williams et al., 2006, Leslie et al., 2009) and lead to metallosis, abnormal peri-prosthetic soft-tissue reactions such as pseudotumours (Kwon et al., 2012). In CoC combinations, edge loading has been associated with accelerated articulating wear, squeaking, stripe wear on either the head or the cup, and in some situations, the fracture of the components (Nevelos et al., 2001, Jarrett et al., 2009, Al-Hajjar et al., 2010). Finite element (FE) studies have also been conducted to examine the stresses in the components due to edge loading and have shown a 3-8 fold increase in the stress of the components in CoC hips compared to that under normal loading conditions (Mak et al., 2002, Sariali et al., 2012). All these studies have provided significant indication that edge loading due to the rotational and translational malposition of the components has a negative impact on the THRs. However, whilst edge loading has been widely investigated for hard-on-hard articulations, fewer studies have been carried out for hard-on-soft combinations, especially with respect to the contact mechanics of modular MoP THR under microseparation conditions. The aim of the present study was therefore to investigate the contact mechanics of a modular MoP THR under edge loading conditions due to the microseparation and rotational malpositioning of the components using FE methods.

## 2 Materials and methods

A typical commercially available modular MoP total hip system, consisting of metal shell, polyethylene liner and metallic femoral head, was analysed. The nominal diameters of the femoral head and inner surface of the polyethylene liner were 36 mm and 36.6 mm respectively, giving a radial clearance of 0.3 mm between the femoral head and the liner. The outer diameter of the acetabular component was assumed to be 54 mm. A polar fenestration with diameter of 20 mm was considered in the central dome region of the metal shell.

**Fig. 1:**
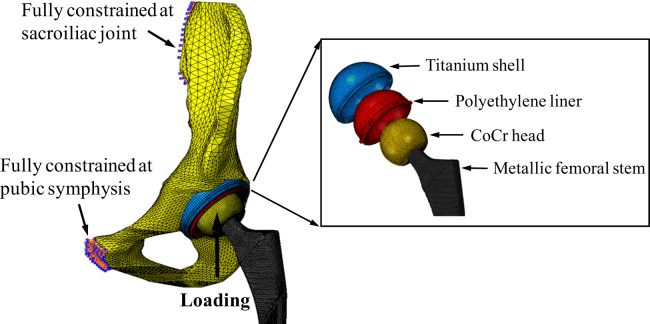
The boundary conditions and components of the finite element model.

A three-dimensional FE model was created to simulate the position of both the femoral and acetabular components implanted in a hemi-pelvic bone model (Fig. 1). The hemi-pelvic bone model consists of a cancellous bone region surrounded by a uniform cortical shell of 1.5 mm thickness (Udofia et al., 2007). The acetabular subchondral bone was assumed to have been reamed completely prior to implantation.

**Fig. 2:**
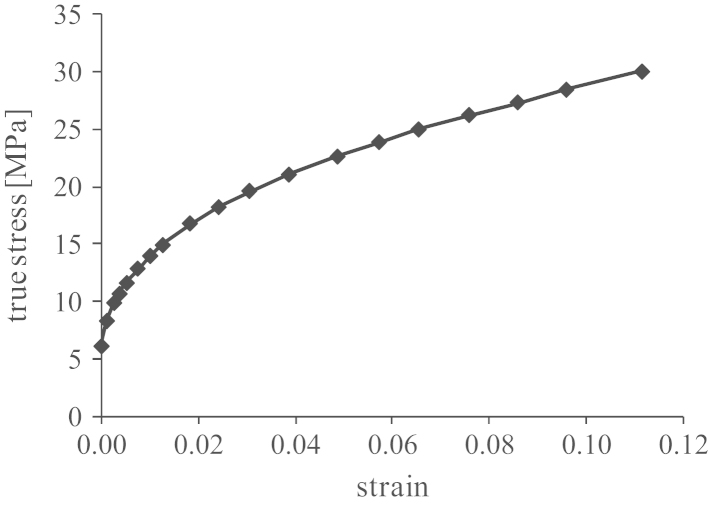
The plastic stress-strain relation for the polyethylene liner (Liu, 2005).

All the materials in the FE model were modelled as homogenous, isotropic and linear elastic except the polyethylene liner which was modelled as non-linear elastic-plastic with the plastic stress-stain constitutive relationship showing in Fig. 2 (Liu, 2005). The mechanical properties for the materials are presented in Table 1 (Udofia et al., 2007, Hua et al., 2012). The femoral component was assumed to be rigid because the elastic modulus of the metallic femoral component is at least two orders of magnitude greater than that of the polyethylene material. The total number of elements for the FE model was approximately 92, 000, predominantly consisting of eight-node brick elements, six-node wedge elements, four-node tetrahedral elements and three-node shell elements. The sensitivity of the results to the mesh was carried out in the cases of standard conditions and 1500 µm microseparation conditions under cup inclination angle of 65°, and results showed that when the number of the elements was doubled, the change in any of the parameters of interest was within 5%.

**Table 1:** The material properties for the components in the present study (Udofia et al., 2007, Hua et al., 2012).

Components	Materials	Young׳s modulus (GPa)	Poisson׳s ratio
Polyethylene liner	UHMWPE	1	0.4
Metal shell	Titanium	116	0.25
Cortical shell	Cortical bone	17	0.3
Cancellous bone	Cancellous bone	0.8	0.2

A sliding contact formulation was used both on the articulating surface and at the metal shell/liner interface, with friction coefficients of 0.083 and 0.15 respectively (Ramero et al., 2007, Amirouche et al., 2008). The nodes situated at the sacroiliac joint and about the pubic symphysis were fully constrained to simulate the sacral and pubic support of the pelvic bone. The interface between the bone and the implant was fully bonded to simulate a situation where the porous sintered coating and in-grown bone were well bonded (Fig. 1). The rotation of the femoral head was fully constrained while the translation was restrained to ensure that the femoral head was only allowed to move along the loading directions. The FE analysis was performed using ABAQUS software package (Version 6.9, Dassault Systèmes Simulia Corp., Providence, United States). The validation of the FE model was presented in detail in a previous study (Hua et al., 2013), which has shown good agreements of contact areas (within 12%) between the FE predictions and the experimental measurements using Leeds Prosim hip joint simulator.

**Fig. 3:**
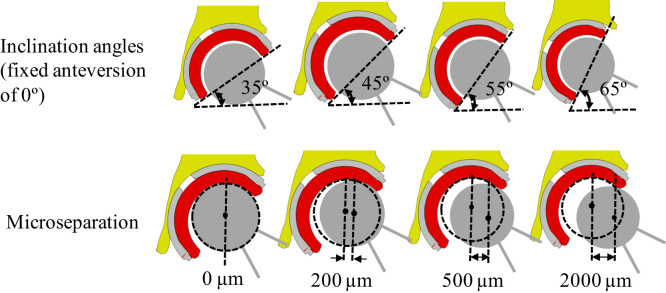
The definition of cup inclination angles and head lateral microseparation distances in the FE model, four cup inclination angles and 12 microseparation distances were considered in the present study. Only four microseparation distances are shown in this figure.

Different loads with magnitude of 2500 N and different directions of 10° medially, 0° (vertical) and 10° laterally were applied through the centre of the femoral head. Four cup inclination angles, varying between 35° and 65° in 10° increments, and 12 lateral microseparation distances of 0 µm, 60 µm, 100 µm, 150 µm, 200 µm, 300 µm, 400 µm, 500 µm, 800 µm, 1000 µm, 1500 µm and 2000 µm were considered in the present study. The definition of the cup inclination angles and head lateral microseparation is shown in Fig. 3.

## 3 Results

**Fig. 4:**
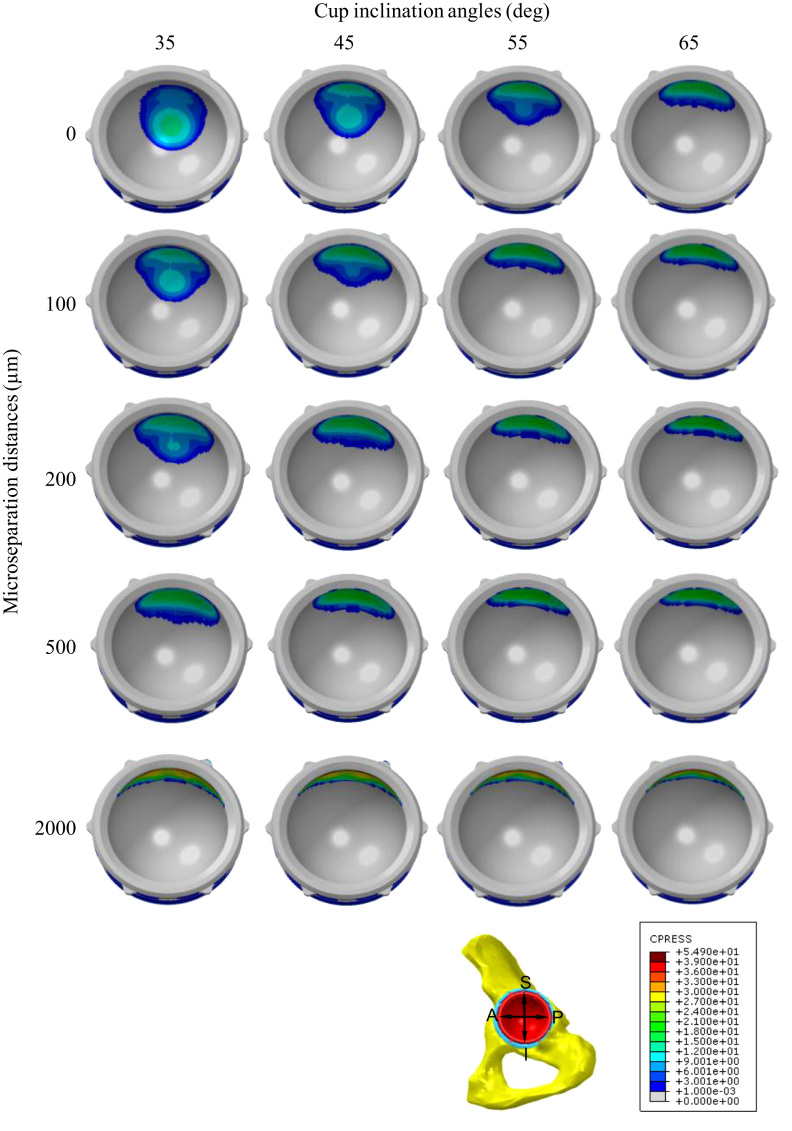
The distribution of contact pressures (MPa) on the frontside articulating surface as a function of cup inclination angles and microseparation distances.

**Fig. 5:**
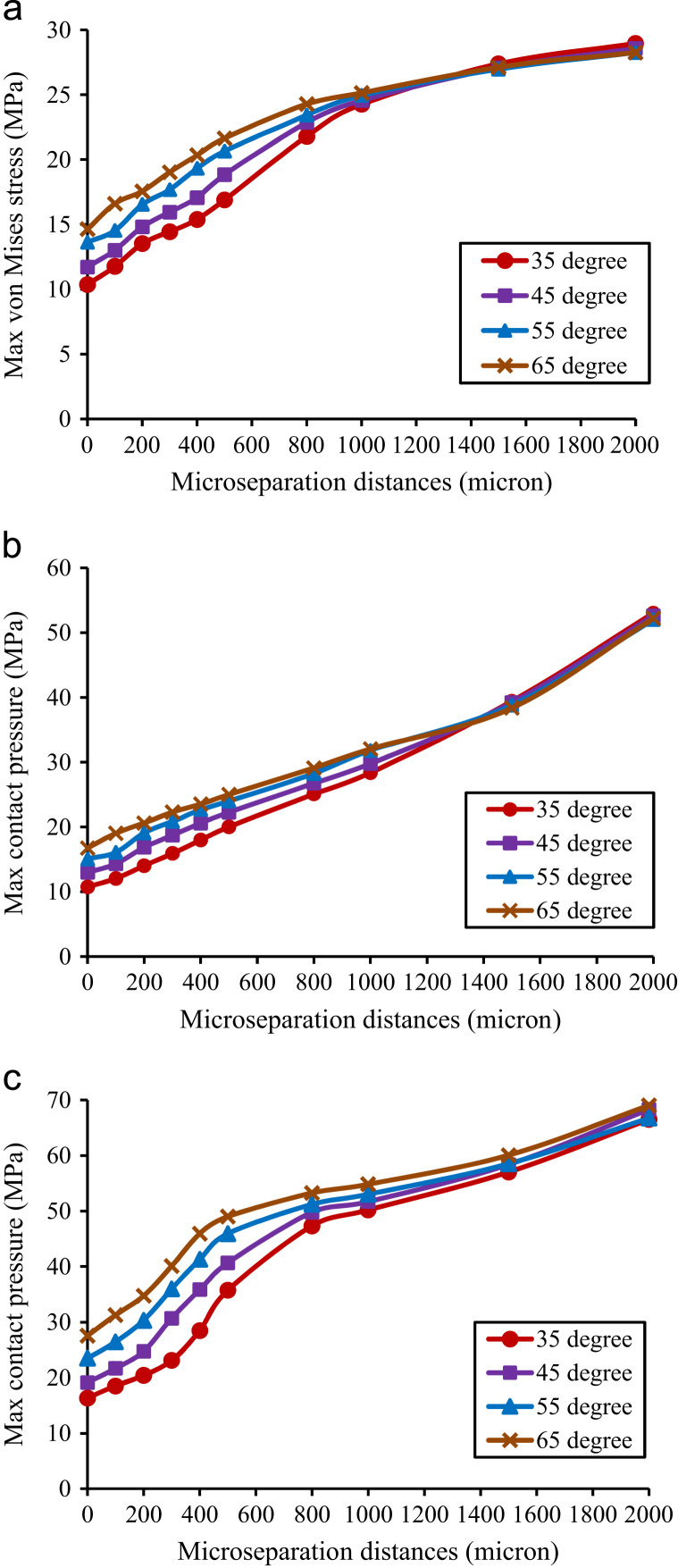
The variation of the maximum stress in the liner against cup inclination angles and microseparation distances: (a) maximum von Mises stress of the liner, (b) maximum contact pressure on the frontside articulating surface, (c) maximum contact pressure on the backside surface of the liner.

Edge loading appeared for lower values of microseparation of the head as the cup inclination angles increased (Fig. 4, Table 2). There was no substantial elevation in the stresses and plastic strain in the liner at the initial occurrence of edge loading. However, the stresses and strain increased continuously when the microseparation distances increased (Fig. 5, Fig. 7).

**Table 2:** The maximum contact pressure (MPa) and edge loading conditions on the articulating surface for different cup inclination angles and microseparation distances.

	Cup inclination angles (deg)			
	35	45	55	65
Microseparation (µm)				
0	10.75	12.98	15.08	16.77
	(no edge contact)	(no edge contact)	(no edge contact)	(no edge contact)
60	11.35	13.96	15.8	18.51
	(no edge contact)	(no edge contact)	(no edge contact)	(edge contact)
100	12.06	14.32	17.09	19.03
	(no edge contact)	(no edge contact)	(edge contact)	(edge contact)
150	12.63	15.86	18.13	19.94
	(no edge contact)	(edge contact)	(edge contact)	(edge contact)
200	14.02	16.86	19.09	20.62
	(edge contact)	(edge contact)	(edge contact)	(edge contact)
300	15.91	18.72	20.84	22.3
	(edge contact)	(edge contact)	(edge contact)	(edge contact)
500	20.02	22.27	24	25.01
	(edge contact)	(edge contact)	(edge contact)	(edge contact)
2000	53.02	52.59	52.21	52.01
	(edge contact)	(edge contact)	(edge contact)	(edge contact)

For all cup inclination angles considered, the maximum von Mises stress of the liner and the maximum contact pressure on the articulating surface and backside surface of the liner increased markedly by 88%–160%, 135%–256%, and 117%–250% respectively when the microseparation distances increased to 2000 µm compared to those under standard conditions (Fig. 5). Under standard conditions, as the cup inclination angles increased, all of the above parameters increased as well. However, the increase of the maximum von Mises stress and contact pressure induced by higher cup inclination angles became negligible as the microseparation distances increased (Fig. 5).

**Fig. 6:**
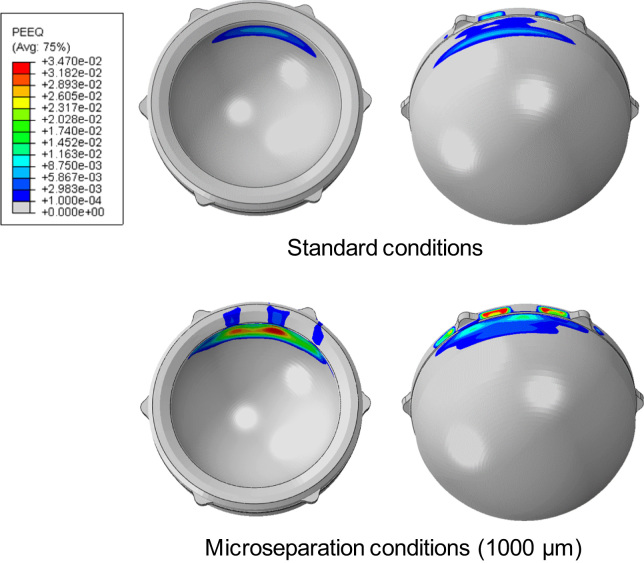
The equivalent plastic strain in the polyethylene liner under standard conditions and microseparation conditions (1000 µm) under cup inclination angle of 45°.

Plastic deformation in the liner was observed under both standard conditions and microseparation conditions. The maximum equivalent plastic strain in the liner was predicted to be 5.6×10^−3^ under standard conditions and increased to 34.7×10^−3^ under 1000 µm microseparation conditions for cup inclination angle of 45° (Fig. 6).

**Fig. 7:**
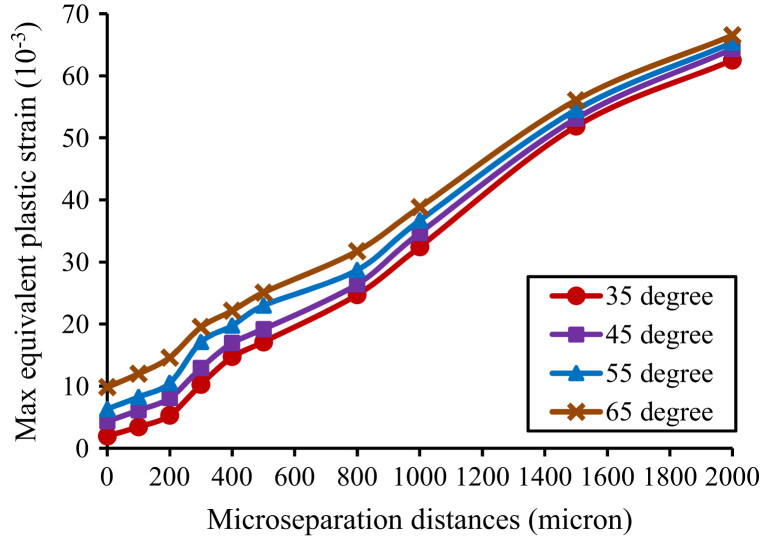
The variation of the maximum equivalent plastic strain in the liner against cup inclination angles and microseparation distances.

For all cup inclination angles considered, the maximum equivalent plastic strain in the liner increased substantially with increased microseparation distances (Fig. 7). The maximum equivalent plastic strain in the liner under microseparation conditions of 2000 µm was predicted to be approximately six times that under standard conditions (Fig. 7).

**Fig. 8:**
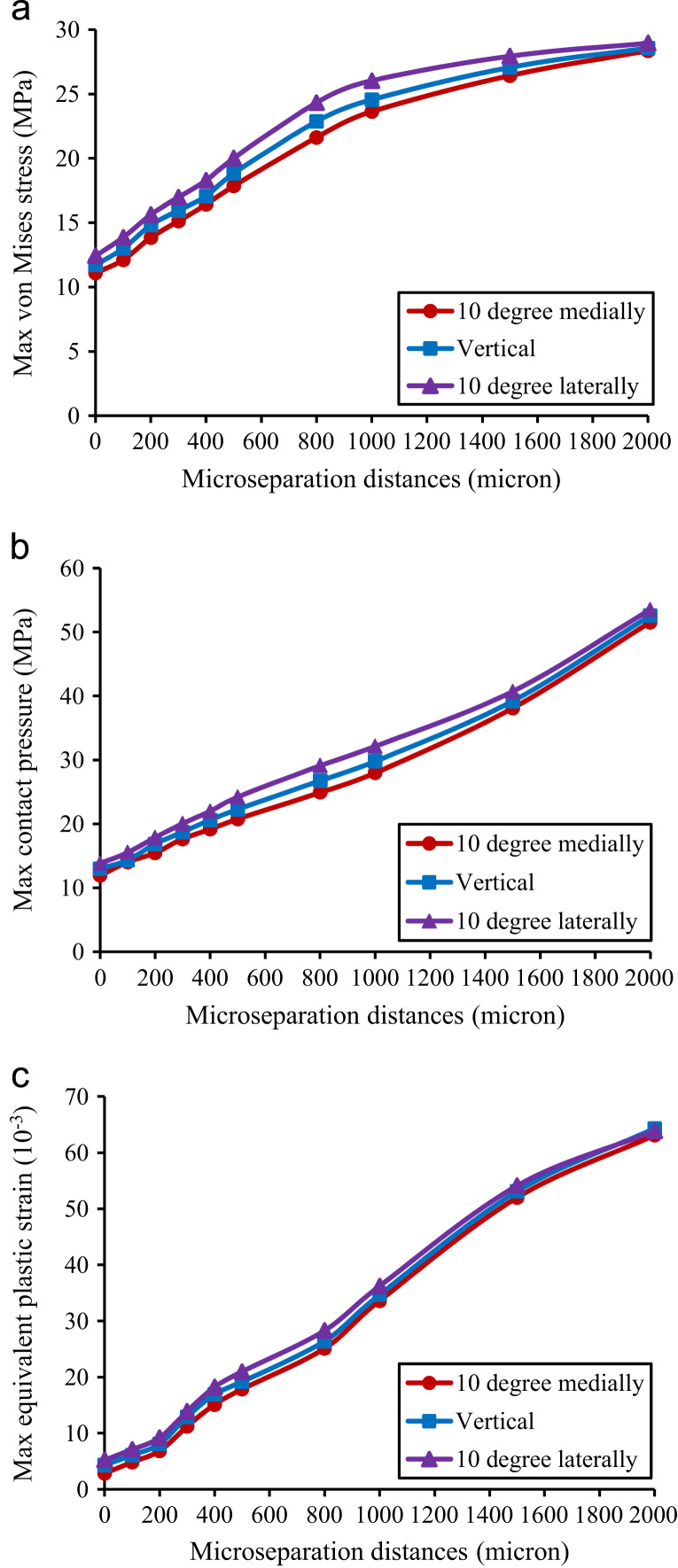
The variation of the maximum stress and equivalent plastic strain in the liner against loading directions and microseparation distances: (a) maximum von Mises stress of the liner, (b) maximum contact pressure on the articulating surface, (c) maximum equivalent plastic strain in the liner.

Under the same microseparation conditions, the model with a load direction of 10° laterally predicted higher maximum von Mises stress, maximum contact pressure and maximum equivalent plastic strain compared to that with a vertical load direction and a load direction of 10° medially (Fig. 8). The discrepancies of these stresses and strain increased from 1.33 MPa, 1.89 MPa and 1.36×10^−3^ under standard conditions to 2.7 MPa, 4.19 MPa and 4.13×10^−3^ under 800 µm microseparation conditions respectively, which then decreased to 0.59 MPa, 1.92 MPa and 0.77×10^−3^ under 2000 µm microseparation conditions (Fig. 8).

## 4 Discussion

Edge loading as an adverse condition which could cause clinical problems has been widely investigated for hard-on-hard bearing designs (Manaka et al., 2004, Williams et al., 2006, Kwon et al., 2012). However, for hard-on-soft combinations, limited work has been undertaken and the effect of edge loading on the behaviour of these articulations deserves further attention. The aims of the present study were therefore to focus on edge loading in a current modular MoP THR, and to examine the effect of cup inclination angles and microseparation on edge loading and contact mechanics of a modular MoP THR.

The FE analysis in the present study showed that with increased cup inclination angles, the microseparation distances required to generate edge loading decreased, suggesting that a steep cup inclination angle could potentially facilitate the occurrence of edge loading when the head lateral microseparation exists. This highlights the increased instability of hip prostheses with steep cups *in vivo* and was found to be consistent with previous clinical studies, indicating that cups positioned with high inclination angles were more likely to suffer from edge loading (Nevelos et al., 1999, Nevelos et al., 2000).

The contact areas on the articulating surface were located about the superior region of the liner under standard conditions, and were found to centralise at the rim of the liner when the microseparation distances increased to 2000 µm, leading to a stripe contact area at the rim of the liner and elevated stresses in this area. This elevation of stresses would cause severe plastic deformation in the liner. Indeed, the FE predictions from this study showed that plastic deformation of the liner occurs under both standard conditions and microseparation conditions. However, there was a substantial increase in the equivalent plastic strain as the microseparation distance increased, indicating severe plastic deformation of the liner under microseparation conditions, which has also been observed in an *in vitro* study (Williams et al., 2003). The severe plastic deformation at the rim of the liner could potentially induce creep and fatigue of the liner (Penmetsa et al., 2006; Hertzberg and Manson, 1980), and also pitting and delamination of the surface at this area, leading to fatigue damage and fracture of the component (Edidin et al., 1999).

The contact stresses on both the frontside articulating surface and backside surface of the liner were found to increase as the cup inclination angles increased. This was consistent with a previous study (Kurtz et al., 1997). However, the differences of contact stresses induced by different cup inclination angles became negligible due to the lateral microseparation of the component. This indicated that in case of hip laxity, the dominating factor to affect the biomechanics of the modular MoP THR is the level of microseparation, rather than acetabular component position. However, the two factors are not independent. It has been shown that clinically a steep cup inclination angle may increase the frequency of occurrence of microseparation at a certain level (Nevelos et al., 1999, Nevelos et al., 2000).

Microseparation is believed to generate elevated both localised wear and global wear for hard-on-hard articulations (Stewart et al., 2001, Leslie et al., 2009, Al-Hajjar et al., 2010). However, it may not be true for hard-on-soft combinations. The limited experimental work to date with ceramic-on-polyethylene (CoP) bearings did not indicate an increase in surface wear when inferior and lateral translations of 0.7 mm were introduced (Williams et al., 2003). However, the present study has shown that the introduction of microseparation to the gait cycle did increase the von Mises stresses in the liner and contact stresses on the articulating surface, and more importantly the plastic strain in the liner. This highlights the importance of the surgical technique in positioning the centre of the head in the centre of the cup to avoid head lateral displacement and thus reduce the maximum stress and strain in the liner component.

The magnitude and orientation of the contact forces were reported to vary over the gait cycle (Bergmann et al., 2001), which were found to affect the biomechanical behaviour of the hip replacements (Kurtz et al., 1997). The present study showed that with a lateral direction of load, the stress and plastic strain in the liner and contact pressure on the articulating surface were predicted to be higher compared to the conditions with a vertical direction and a medial direction of loads. This phenomenon was found to be aggravated under moderate microseparation conditions (i.e. 500 µm and 800 µm microseparation conditions) where the discrepancies of predictions of stresses and plastic strain among the models with different load directions were 2–3 times that under standard conditions. This again highlights the importance of the surgical technique in avoiding the head lateral microseparation in clinical practice.

There are a number of limitations to this study. First of all, the soft tissues surrounding the hip such as muscles and ligaments may have an important role in the stability of the hip replacement (Elkins et al., 2011). These were not considered in the current study. Secondly, the head lateral microseparation during gait is actually a dynamic process (Lombardi et al., 2000, Dennis et al., 2001). In addition, the magnitude and orientation of the contact forces varied over gait cycle. However, in the present study, a static analysis with fixed load was performed and the effect of dynamic impact on the stresses and strain was not explicitly considered. Nevertheless, a contact force of 2500 N was applied in the present study, which was higher than physiological loading during gait (Bergmann et al., 2001) and can be expected to offset the dynamic impact. Different load directions of 10° medially and 10° laterally as well as a vertical load were also considered under microseparation conditions in the present study to simply represent the different load directions during gait. Even so, the dynamic process of microseparation during gait and exact physiological loading conditions should be simulated and addressed in the models in future studies. Additionally, the femoral head was loaded at its centre in the present study, without considering an off-centre location. However, as the femoral head was assumed to be rigid, the models would predict identical results with respect to the contact pressure on the bearing surface and the plastic strain of the liner irrespective of the location of applied loading on the femur. Moreover, the material properties of the liner used in the present study was suitable for conventional UHMWPE. However, for current MoP hip replacement, there is a higher usage of cross-linked UHMWPE, which has a 2–3 times higher Young׳s modulus compared to the conventional UHMWPE (Lewis, 2001). In order to examine the effect of material properties, sensitivity analysis of model predictions to UHMWPE material properties were conducted and the results showed that when the Young׳s modulus of UHMWPE changed from 1 GPa–2.5 GPa, the maximum contact pressures and equivalent plastic strain in the liner increased by about 5% and 4% respectively under standard conditions, and about 7% and 8% respectively under 1000 µm microseparation conditions.

Another limitation in the present simulation was that the interface between the bone and metal shell was fully bonded to simulate a solid bone-implant osseointegration situation. However, in reality, it is impossible to ensure perfect integration between the bone and the prosthesis with current surgical techniques, especially in the early stage of implantation. In order to assess the effect of such assumption on the predicted results, additional simulations considering a frictional contact interface between the bone and the implant were conducted. The simulation results showed that the contact pressures at the articulating surface and the equivalent plastic strain of the liner decreased by about 13.4% and 17.3% when a friction coefficient of 0.6 (Ramamurti et al., 1997; Mischler and Pax, 2002) was considered at the bone/metal shell interface under microseparation conditions of 1000 µm. In addition, a certain degree of micromotion (about 38 µm) for the implants was predicted at this situation. This micromotion would contribute to the loosening and instability of the implants, especially at the primary stage of implantation.

Despite these limitations listed above, this study did suggest that the head lateral microseparation would cause edge loading and induce a marked increase in the von Mises stresses of the liner and contact stresses on the articulating surfaces, as well as severe plastic deformation of the liner, and that steep cup inclination angles would facilitate edge loading. Therefore, clinically it is important to avoid conditions that may lead to edge loading, which means reducing the levels of rotational and translational mal-positioning of the head and cup.

## Conflict of interest statement

John Fisher is a consultant to DePuy Synthes Joint Reconstruction.
